# Identification of the Potential Virulence Factors and RNA Silencing Suppressors of Mulberry Mosaic Dwarf-Associated Geminivirus

**DOI:** 10.3390/v10090472

**Published:** 2018-09-03

**Authors:** Xiuling Yang, Yanxiang Ren, Shaoshuang Sun, Dongxue Wang, Fanfan Zhang, Dawei Li, Shifang Li, Xueping Zhou

**Affiliations:** 1 State Key Laboratory for Biology of Plant Diseases and Insect Pests, Institute of Plant Protection, Chinese Academy of Agricultural Sciences, Beijing 100193, China; xlyang@ippcaas.cn (X.Y.); sz20163020161@cau.edu.cn (Y.R.); sunshaoshuangsss@163.com (S.S.); wwangdx@163.com (D.W.); 21716078@zju.edu.cn (F.Z.); 2 State Key Laboratory of Agro-Biotechnology and Ministry of Agriculture Key Laboratory of Soil Microbiology, College of Biological Sciences, China Agricultural University, Beijing 100193, China; lidw@cau.edu.cn; 3 State Key Laboratory of Rice Biology, Institute of Biotechnology, Zhejiang University, Hangzhou 310058, Zhejiang, China

**Keywords:** MMDaV, RNA silencing, suppressor, virulence factor

## Abstract

Plant viruses encode virulence factors or RNA silencing suppressors to reprogram plant cellular processes or to fine-tune host RNA silencing-mediated defense responses. In a previous study, Mulberry mosaic dwarf-associated virus (MMDaV), a novel, highly divergent geminivirus, has been identified from a Chinese mulberry tree showing mosaic and dwarfing symptoms, but the functions of its encoded proteins are unknown. In this study, all seven proteins encoded by MMDaV were screened for potential virulence and RNA silencing suppressor activities. We found that V2, RepA, and Rep affect the pathogenicity of a heterologous potato virus X. We showed that V2 could inhibit local RNA silencing and long-distance movement of the RNA silencing signal, but not short-range spread of the green fluorescent protein (GFP) silencing signal in *Nicotiana benthamiana* 16c plants. In addition, V2 localized to both subnuclear foci and the cytoplasm. Deletion mutagenesis of V2 showed that the basic motif from amino acids 61 to 76 was crucial for V2 to form subnuclear foci and for suppression of RNA silencing. Although the V2 protein encoded by begomoviruses or a curtovirus has been shown to have silencing suppressor activity, this is the first identification of an RNA silencing suppressor from a woody plant-infecting geminivirus.

## 1. Introduction

Viruses are intracellular obligate parasites that absolutely depend on the host machinery for their replication and movement. To defend themselves against invading viruses, plants employ several layers of immune responses [1,2,3,4,5]. RNA silencing, a fundamental sequence-specific gene regulatory process, has been demonstrated to be one of the major antiviral defense mechanisms in plants [3,6]. Briefly, viral double-stranded RNA (dsRNA) molecules of different origins, namely highly structured regions of viral single-stranded RNAs, replicative intermediates, or overlapping bidirectional read-through transcripts can trigger RNA silencing, and these are processed into 21–24 nucleotides (nt) small interfering RNAs (siRNAs) by Dicer-like RNases (DCLs). The siRNAs are stabilized through HUA Enhancer 1 (HEN1)-dependent 2′-O-methylation at their 3′ end, and incorporated into an Argonaute (AGO)-containing RNA-induced silencing complex (RISC) to cause sequence-specific degradation of target RNAs, or into RNA-induced transcriptional silencing complex (RITS) to induce histone and/or DNA methylation. In plants, the antiviral silencing signal can be amplified by RNA-dependent RNA polymerases (RDRs). Finally, siRNAs can act as mobile silencing signals to trigger local silencing upon movement from cell-to-cell and systemic silencing, following their transport through phloem tissues [3].

To achieve a successful infection, plant viruses encode proteins, which are referred to as viral suppressors of RNA silencing (VSRs), to thwart the antiviral RNA silencing machinery [7]. The VSRs examined to date were shown to target virtually all steps of the RNA silencing pathway, such as the silencing initiation phase, the effector phase, and the amplification phase [6]. For example, the P19 protein encoded by tombusviruses [8,9,10], HC-Pro from several potyviruses [11,12], and P21 from beet yellows virus [13], block the silencing initiation step by sequestration of siRNAs, while the P0 protein of *Polerovirus* was shown to promote AGO1 degradation [14,15]. Many VSRs, such as the 2b protein of cucumber mosaic virus (CMV) [16,17] or P38 of turnip crinkle virus [18,19], are able to target multiple steps of the RNA silencing pathway. Available evidence suggests that the identified VSRs within and across kingdoms are highly diverse in their sequences, structures, and modes of action. In addition to suppressing RNA silencing, most of the VSRs are also responsible for other functions during the viral infection, such as symptom induction, replication, and cell-to-cell movement. The great diversity and multifunctional characteristics of VSRs reinforces the importance of the identification of new RNA silencing suppressors, and the elucidation of their interplay with the plant RNA silencing machinery.

Geminiviruses are important plant DNA viruses that infect a wide range of crops in tropical and subtropical regions. By taking advantage of the small RNA-based deep sequencing technology [20], new geminivirus species have been discovered in the past few years. Our knowledge about the host range of geminiviruses in nature is expanding, and it is now clear that these viruses infect not only herbaceous plants, but also woody plants, including citrus, grapevine, mulberry, and apple trees [21,22,23,24,25]. Based on their genome structures, insect vectors and host range, geminiviruses are currently classified into nine genera (*Becurtovirus*, *Begomovirus*, *Capulavirus*, *Curtovirus*, *Eragrovirus*, *Grablovirus*, *Mastrevirus*, *Topocuvirus*, and *Turncurtovirus*) [26]. With limited coding capacities, geminiviruses are reported to redirect and reprogram multiple plant processes [27]. Independent studies have shown that geminiviruses are both inducers and targets of the host antiviral RNA silencing response. Since the first description of the begomovirus C2 protein as a VSR, C4/AC4, V2/AV2, and Rep encoded by different species of geminiviruses, and βC1 encoded by the begomovirus-associated betasatellite, have been reported to suppress RNA silencing at the posttranscriptional level (posttranscriptional gene silencing, PTGS), and/or at the transcriptional level (transcriptional gene silencing, TGS) in different manners [6,27,28].

Mulberry mosaic dwarf-associated geminivirus (MMDaV) is a distinct monopartite geminivirus that is found in Chinese mulberry trees showing mosaic and dwarfing symptoms [25]. It has a monopartite genome of 2952 nucleotides, encoding five open reading frames (ORFs, V1, V2, V3, V4, and V5) on the virion-sense strand, and two ORFs (C1 and C2) on the complementary-sense strand. V1, V2, C1, and C2 of MMDaV share the highest sequence identities with the cognate coat protein (CP), movement protein, Replication-associated protein (Rep), and RepA-like proteins of citrus chlorotic dwarf associated virus. However, V3, V4, and V5 show no significant homologies to any other proteins reported in GenBank [25]. Due to its recent characterization and difficulties in the study of woody plant-virus interactions, no information on the functions of MMDaV-encoded proteins is currently available. In this study, we screened for the potential virulence factors and RNA silencing suppressors encoded by MMDaV. We found that V2, RepA, and Rep affect the pathogenicity of a heterologous potato virus X (PVX). We also show that the V2 protein is able to suppress the host RNA silencing machinery.

## 2. Materials and Methods

### 2.1. Plant Materials

*Nicotiana benthamiana* plants, and *N. benthamiana* line 16c, which transgenically expresses a green fluorescent protein (GFP) [29], were grown from seeds in an insect-free growth room at 25 °C under a 16:8 h (light/dark) photoperiod.

### 2.2. Generation of Plasmid Constructs

The six ORFs (V1, V2, V3, V5, V4, and RepA) encoded by MMDaV were individually amplified from rolling circle amplification products of the AK2 isolate of MMDaV (GenBank accession no. KP303687) [25] by polymerase chain reaction (PCR), using specific primers that contained suitable restriction sites (Table 1). The Rep ORF was amplified from the complementary DNA (cDNA) of the AK2 isolate. PCR products were individually cloned into the pGEM-T Easy vector (Promega, Madison, WI, USA) to generate pGEM-T-V1, pGEM-T-V2, pGEM-T-V3, pGEM-T-V4, pGEM-T-V5, pGEM-T-RepA, and pGEM-T-Rep, which were individually digested with specific enzymes for subsequent cloning. To test for pathogenicity, each ORF was cloned into the PVX-containing pgR106 vector (a kind gift from David C. Baulcombe, University of Cambridge, Cambridge, UK) between the *Cla*I and *Sal*I restriction sites to yield PVX-V1, PVX-V2, PVX-V3, PVX-V4, PVX-V5, PVX-RepA, and PVX-Rep, respectively. The resulting recombinant PVX constructs were individually transformed into the *Agrobacterium tumefaciens* strain GV3101 by electroporation.

For the PTGS suppression assay, the full-length ORF of V1 was subcloned into the pCHF3 vector [30] between the *Sma*I and *Sal*I sites to produce pCHF3-V1. The full-length ORFs of V2, V3, RepA, and Rep were individually subcloned into the pCHF3 vector between the *BamH*I and *Sal*I sites, to yield pCHF3-V2, pCHF3-V3, pCHF3-RepA and pCHF3-Rep. The full-length ORFs of V4 and V5 were individually subcloned into the pCHF3 vector between the *Kpn*I and *Sal*I sites, to obtain pCHF3-V4 and pCHF3-V5. The resulting constructs were individually introduced into the *A. tumefaciens* strain C58C1 through electroporation.

For subcellular localization analysis, the full-length fragment of MMDaV V2 was inserted into the *BamH*I and *Sal*I sites of the pCHF3-N-eGFP vector [31] to produce 35S-GFP-V2, which contains a V2 N-terminal fusion protein with enhanced green fluorescent protein (eGFP). The resulting plasmid was mobilized into *A. tumefaciens* strain C58C1 via electroporation.

To generate the V2 mutant dm61-77aa, the 61RRLLRLIRRFSRVKDR76 motif was deleted from the plasmid pGEM-T-V2, using KOD-Plus-Mutagenesis Kit as instructed (Toyobo, Osaka, Japan). The resulting V2^dm61-77aa^ was inserted into the *BamH*I and *Sal*I sites of pCHF3 or pCHF3-N-eGFP to produce pCHF3-V2^dm61-77aa^ and 35S-GFP-V2^dm61-77aa^, respectively. All primers used for the generation of the DNA constructs described above are shown in Table 1.

### 2.3. Agrobacterium-Mediated Virus Inoculation and Transient Gene Expression

Agrobacterium-mediated virus inoculation or transient gene expression were carried out as described [32]. *Agrobacterium* cultures were pelleted and resuspended to an optical density OD_600_ = 1.0 in a solution containing 10 mM MgCl_2_, 10 mM MES (pH 5.8), and 100 μM acetosyringone, and were incubated at room temperature for 2–3 h prior to infiltration.

For the recombinant PVX vectors expressing individual ORFs of MMDaV, cultures of *A. tumefaciens* harboring different constructs were individually infiltrated into *N. benthamiana* leaves at an *OD*_600_ = 1.0.

To test silencing suppression activity, equal volumes of *Agrobacterium* cultures harboring 35S-GFP (a 35S promoter-driven construct expressing sense GFP, a kind gift from David C. Baulcombe, University of Cambridge, Cambridge, UK) and the tested constructs were mixed, followed by infiltration into fully expanded leaves of four-week-old 16c or wild type *N. benthamiana* plants. Co-infiltration of 35S-GFP with a construct to express tomato bushy stunt virus (TBSV) P19 was used as a positive control, and that with the empty vector of pCHF3 was used as a negative control. For dsRNA-induced PTGS experiments, *A. tumefaciens* cultures containing the 35S-GFP, 35S-dsGFP (a construct expressing an inverted repeat sequence of GFP) [33], together with either pCHF3-V2, or P19, or the empty pCHF3 vector, were mixed in equal proportions and infiltrated into *N. benthamiana* 16c plant leaves. GFP fluorescence in infiltrated or systemic leaves was monitored under handheld long-wavelength UV lamp (Black-Ray Model B-100A, San Gabriel, CA, USA) and photographed with a Canon EOS 70D digital camera mounted with a 58 mm yellow filter.

For subcellular localization analysis, *A. tumefaciens* cultures harboring pCHF3-eGFP, 35S-GFP-V2, or 35S-GFP-V2^dm61-76aa^ was infiltrated into four-week-old *N. benthamiana* plants as described [31].

### 2.4. RNA Extraction and Analysis

Total RNA was extracted using TRIzol reagent following the manufacturer’s instructions (Invitrogen, Carlsbad, CA, USA). For the *N. benthamiana* plants inoculated with PVX or recombinant PVX constructs, systemically infected plant leaves were harvested and prepared to analyze MMDaV ORF expression by reverse transcription PCR (RT-PCR). 1 μg of total RNA was reverse transcribed into cDNA using a PrimeScript RT reagent Kit with genomic DNA Eraser (Takara, Dalian, China). Expression of the individual MMDaV ORFs was detected by PCR using primers listed in Table 1. For GFP messenger RNA (mRNA) analysis, 10 μg of total RNA extracted from infiltrated patches of *N. benthamiana* line 16c plants was separated on 1.2% formaldehyde-agarose gels and transferred to Hybond N+ membranes as instructed (GE Healthcare, Buckinghamshire, UK). The membrane was hybridized with a digoxigenin-labeled GFP probe, which was made using the PCR DIG probe synthesis kit, and was detected using a detection starter kit II according to the manufacturer’s instructions (Roche Diagnostic, Basel, Switzerland).

### 2.5. Protein Extraction and Western Blot Analysis

Extraction of total soluble proteins, SDS-PAGE, and Western blot analysis were performed as described [31]. For detection of PVX, proteins were extracted from systemically infected leaves of *N. benthamiana* plants infected with PVX or PVX recombinant constructs. The anti-CP monoclonal antibody (MAb) raised against PVX (raised in our lab, Institute of Biotechnology, Zhejiang Univiersity, Hangzhou, China) was used at a 1:8000 dilution. For detection of GFP, proteins were extracted from infiltrated patches of *N. benthamiana* line 16c or *N. benthamiana* plants. The anti-GFP MAb (Roche) was used at a 1:8000 dilution. Western blots were visualised with a secondary peroxidase-conjugated goat antimouse antibody (Cell Signaling Technology, Boston, MA, USA) and a chemiluminescence detection system (Tianneng, Shanghai, China).

### 2.6. Laser-Scanning Confocal Microscopy

Imaging of fluorescent proteins was conducted using a confocal microscopy (LSM880; Carl Zeiss, Jena, Germany) at 36 to 48 h post-infiltration. To stain the nuclei of the leaf epidermal cells, 0.1 μg·mL^−1^ of 4′-6-diamidino-2-phenylindole (DAPI) was infiltrated into *N. benthamiana* leaves for 10 to 20 min prior to mounting onto slides as described [34]. For GFP, excitation was set at 488 nm, and emission was set at 500 to 530 nm. For DAPI, excitation was set at 405 nm, and emission was set at 440 to 475 nm.

## 3. Results

### 3.1. Identification of Virulence Factors Encoded by MMDaV

**Figure 1:**
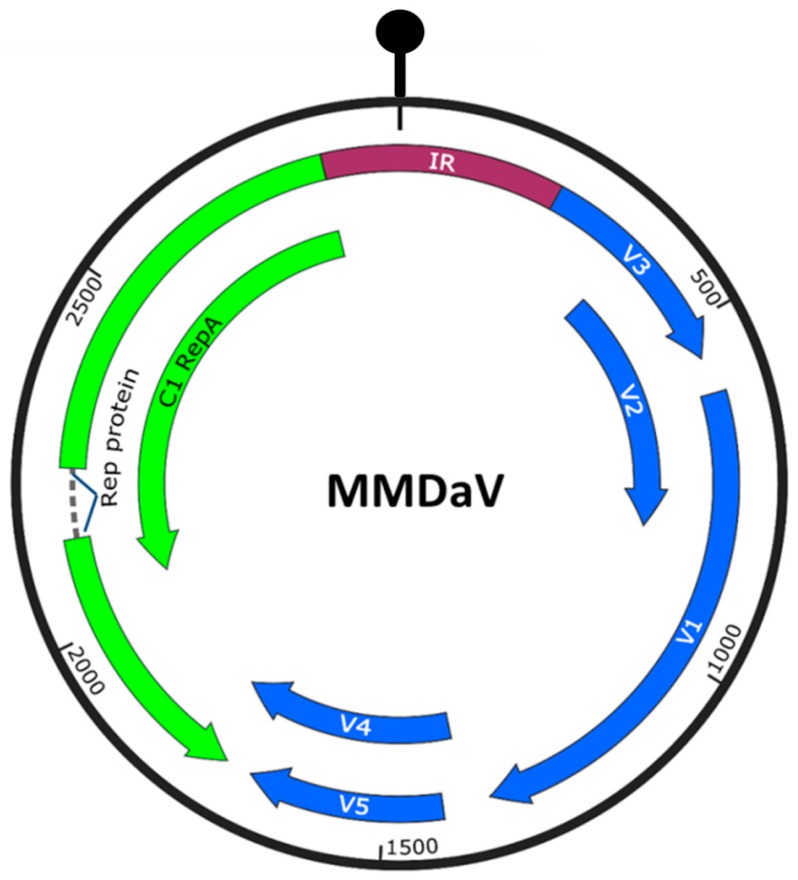
Genome organization of Mulberry mosaic dwarf-associated geminivirus (MMDaV), showing the open reading frames (ORFs) coded. ORFs encoded on the virion-sense (V) strand and complementary-sense (C) strand are denoted as blue and green colors, respectively. The circle represents the circular, single-stranded DNA of MMDaV. The stem-loop structure that contained the conserved nonanucleotide sequence, TAATATTAC, within the intergenic region (IR) is shown on top of the diagram.

**Figure 2:**
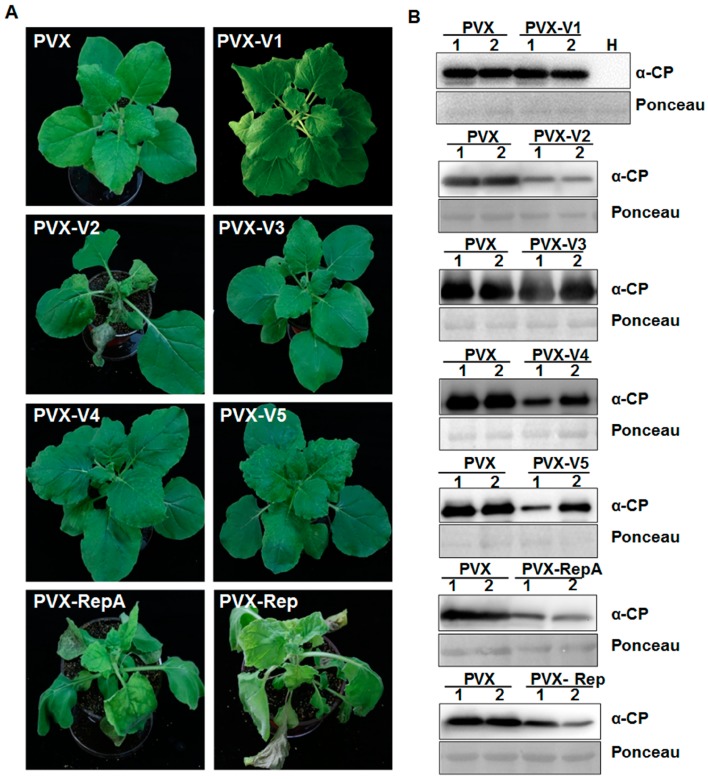
Effects of MMDaV-encoded proteins on potato virus X (PVX) pathogenicity. (**A**) Symptoms of *Nicotiana benthamiana* plants inoculated with *Agrobacterium* cells harboring PVX alone, or recombinant PVX vector expressing individual ORFs of MMDaV. Photographs were taken at 10 days post infiltration (dpi). Experiments were repeated three times, and at least four to six plants were used for each inoculation. (**B**) Western blot analysis of PVX accumulation in inoculated *N. benthamiana* plants at 7 to 10 dpi using a monoclonal antibody against PVX CP. Total proteins were extracted from upper non-inoculated leaves as indicated in (**A**). Two independent plants were used to extract total proteins. H represents total soluble proteins extracted from the healthy *N. benthamiana* plant, which was used to detect the specificity of the PVX antibody. Ponceau staining of the large subunit of Rubisco served as loading controls.

MMDaV has a monopartite genome with seven ORFs (Figure 1). To determine if any of the seven proteins is related to viral pathogenicity, we took advantage of the PVX-based heterologous gene expression system to express the different MMDaV proteins in planta. Leaves of four-week old *N. benthamiana* plants were inoculated with *Agrobacterium* cultures containing PVX or recombinant PVX constructs expressing individual MMDaV ORF and were monitored for symptom development. At seven days post infiltration (dpi), non-inoculated systemic leaves exhibited mosaic symptoms characteristic of PVX infection in *N. benthamiana* plants inoculated with PVX, PVX-V1, PVX-V3, PVX-V4, and PVX-V5, respectively. By 10–12 dpi, *N. benthamiana* plants inoculated with PVX-V4 and PVX-V5 developed milder mosaic symptoms (Figure 2A). However, PVX expressing V2 developed necrosis symptoms in inoculated leaves at 5 dpi, followed by downward leaf curling symptoms in emerging leaves at 7 dpi, and death of the apical shoots and eventual plant death at 11 dpi (Figure 2A). *N. benthamiana* plants inoculated with PVX-RepA showed necrotic lesions in the inoculated leaves at 4 dpi, followed by downward leaf curling at 6 dpi. Collapse of developing tissue was also observed in emerging leaves inoculated with PVX-RepA at 10 dpi (Figure 2A). PVX-Rep induced upward leaf curling at 6 dpi, followed by the collapse of developing leaves at 10 dpi (Figure 2A).

To confirm the stability of the inserts, RT-PCR was performed on total RNA extracted from non-inoculated systemic leaves at 7–10 dpi by using specific primers targeting the individual MMDaV ORFs. RT-PCR amplification on each sample obtained expected fragments from *N. benthamiana* plants infected with recombinant PVX constructs, but not from plants inoculated with PVX, suggesting the maintenance of the MMDaV sequences in the systemically infected leaves. Western blot analysis of the proteins extracted from systemically infected leaves using antibody against PVX CP confirmed PVX infection in all of the inoculated *N. benthamiana* plants (Figure 2B). Furthermore, the presence of V2, RepA, and Rep decreased the accumulation of PVX in non-inoculated systemic leaves. Although the expression of V4 and V5 proteins did not enhance the pathogenicity of PVX, their expression also decreased PVX accumulation (Figure 2B). In summary, the V2, RepA, and Rep proteins of MMDaV enhance the infection severity of a heterologous PVX.

### 3.2. Identification of Suppressors of Local RNA Silencing

**Figure 3:**
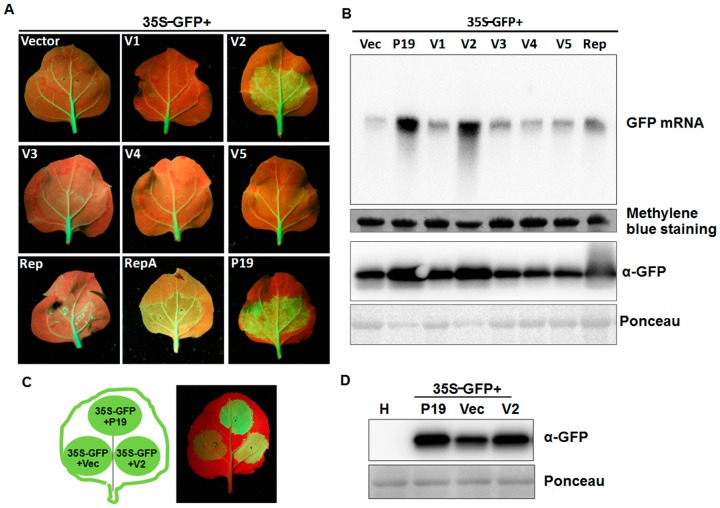
Effects of MMDaV-encoded proteins on GFP local silencing. (**A**) *N. benthamiana* 16c plants were infiltrated with a mixture of *Agrobacterium* cultures containing 35S-GFP and pCHF3 vectors expressing individual ORFs of MMDaV, respectively. The constructs used for infiltration are indicated. *N. benthamiana* 16c plants infiltrated with 35S-GFP, plus the empty vector or 35S-GFP plus tomato bushy stunt virus P19 were used as negative or positive controls, respectively. Photographs were taken under UV light with a yellow filter-mounted Canon camera at 5 dpi. Similar results were obtained in three dependent experiments. At least five plants were agroinfiltrated per experiment. (**B**) Analysis of the GFP mRNA and protein levels in infiltrated leaf patches. 10 μg of total RNA extracted from infiltrated patches at 5 dpi were used in Northern blot analysis. The GFP mRNA was detected using a DIG-labeled *GFP*-specific probe. Methylene blue staining was used to visualize the loading controls for the mRNA. The expression of the GFP protein was analyzed by Western blot using a monoclonal antibody against GFP. Ponceau staining of the large subunit of Rubisco served as loading controls for the Western blot assay. (**C**) Suppression of local post-translational gene silencing (PTGS) in wild-type *N. benthamiana* plants. *N. benthamiana* plants were infiltrated with a mixture of *Agrobacterium* cultures containing constructs as indicated in the left panel. Expression of 35S-GFP with the empty vector or 35S-GFP with P19 served as negative or positive controls, respectively. Photographs were taken under UV light at 3 dpi. (**D**) Western blot analysis of the GFP protein levels in infiltrated *N. benthamiana* leaf patches using a GFP monoclonal antibody. Total soluble proteins extracted from the healthy *N. benthamiana* plants were used to detect the specificity of the GFP antibody. Ponceau staining of the large subunit of Rubisco protein are shown as loading controls.

Discovery of MMDaV by small RNA-based deep sequencing [25] suggested the pivotal role of RNA silencing in targeting MMDaV in mulberry trees. To test whether any of the MMDaV ORFs is able to counteract the host RNA silencing response, a classical GFP-based two-component agroinfiltration assay was used to screen potential VSRs. In this system, sequences encoding the seven ORFs were individually cloned into a binary vector (pCHF3) under the control of a 35S promoter. A mixture of *Agrobacterium* containing 35S-GFP and plasmids expressing individual ORFs of MMDaV was co-infiltrated into fully expanded leaves of *N. benthamiana* GFP line 16c plants. As expected, strong GFP fluorescence was evident in all leaf patches agroinfiltrated with a mixture of 35S-GFP, either with pCHF3 empty vector or pCHF3 vectors expressing individual MMDaV ORFs, at 2 to 3 dpi. At 5 dpi, as a consequence of GFP local silencing, GFP fluorescence was completely lost in the patches agroinfiltrated with 35S-GFP, plus the empty pCHF3 vector. As previously described, agroinfiltration of 35S-GFP and TBSV P19, a positive control for silencing suppression, elicited a strong GFP fluorescence in the infiltrated area at 5 dpi. Examination of the GFP fluorescence in leaf patches co-infiltrated with *Agrobacterium* mixtures containing 35S-GFP and the tested MMDaV ORFs showed that only fluorescence in the case of V2 was comparable with that of P19 at 5 dpi (Figure 3A), which could last even at 8 dpi (Figure S1). None of the other ORFs evaluated had a detectable effect on GFP expression (Figure 3A), nor on the prevention of GFP silencing by 8 dpi (Figure S1). Interestingly, agroinfiltration of 35S-GFP with the RepA or Rep ORF of MMDaV caused necrotic lesions in infiltrated leaf patches (Figure 3A and Figure S1), suggesting the role of RepA and Rep in pathogenicity that may be independent of silencing suppressor activity. As the severe necrosis of the patches caused by infiltration of 35S-GFP plus RepA did not allow for the extraction of RNA of good quality, Northern blot analysis was carried out using total RNAs extracted from leaf patches agroinfiltrated with 35S-GFP plus the empty vector, P19, or the other six ORFs (V1, V2, V3, V4, V5, and Rep). Consistent with the observed GFP fluorescence, GFP mRNA accumulated to higher levels in leaves expressing V2 and P19 than in those transformed with the empty vector or the other ORFs (Figure 3B). Protein gel blots showed that GFP protein was low in leaves co-infiltrated with 35S-GFP together with the empty vector or the other five ORFs. In contrast, higher GFP protein levels were detected in leaves co-infiltrated with 35S-GFP and P19, or with 35S-GFP and V2 (Figure 3B). Similar transient assays were carried out in wild-type *N. benthamiana* plants by infiltration with *Agrobacterium* carrying 35S-GFP and empty vector, or 35S-GFP and V2 or 35S-GFP and P19. As expected, GFP fluorescence faded at 3 dpi in the infiltrated area in control conditions. However, tissues infiltrated with 35S-GFP and V2 or with 35S-GFP and P19 showed strongly increased GFP fluorescence (Figure 3C). Concurrently, levels of GFP protein determined by Western blot analysis correlated with the visualized GFP fluorescence (Figure 3D). These results suggest that MMDaV V2 is a potent suppressor of local RNA silencing triggered by sense GFP.

Since dsRNA is considered to be a strong inducer of RNA silencing [35], we examined whether MMDaV V2 was able to suppress dsRNA-induced RNA silencing in *N. benthamiana* 16c plants. GFP fluorescence was only observed in co-infiltrations that contained 35S-GFP, 35S-dsGFP, and P19 at 3 dpi, but not in leaf patches infiltrated with 35S-GFP, 35S-dsGFP, and empty vector or in those infiltrated with 35S-GFP, 35S-dsGFP, and MMDaV V2 (Figure S2), suggesting that MMDaV V2 does not suppress dsRNA-induced RNA silencing.

### 3.3. MMDaV V2 Does Not Suppress Cell-to-Cell Movement of Silencing Signal

**Figure 4:**
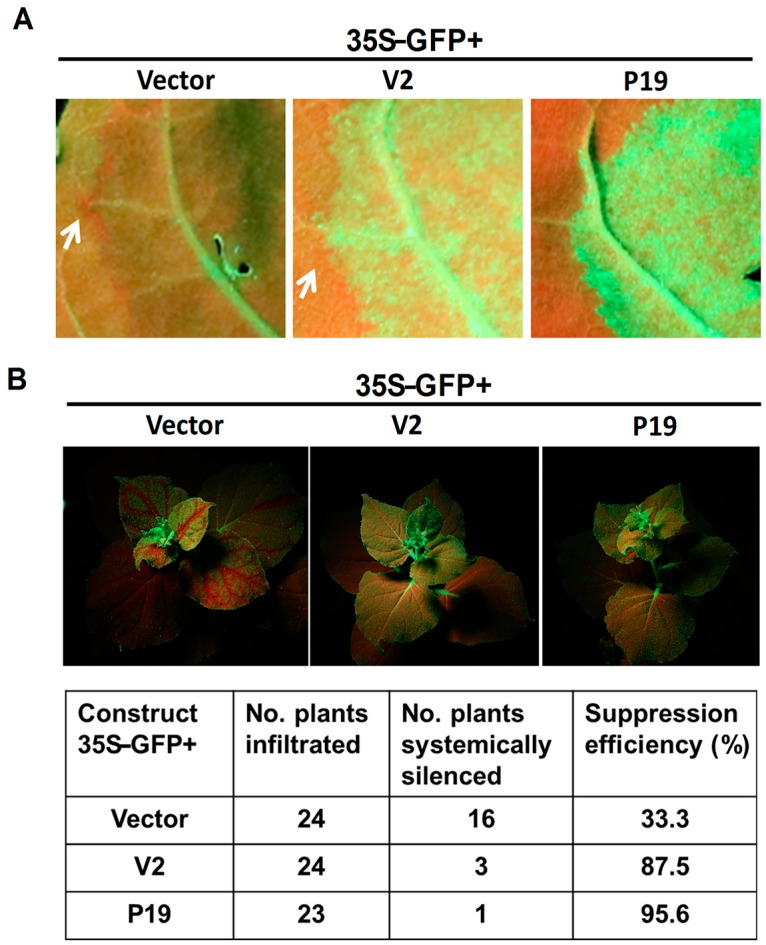
Effect of MMDaV V2 on short- and long-range spread of the silencing signals. (**A**) MMDaV V2 could not inhibit short-distance spread (10–15 cells) of the GFP silencing signal in *N. benthamiana* 16c plants. Leaves were infiltrated with a mixture of *Agrobacterium* cultures containing 35S-GFP and MMDaV V2. Leaves infiltrated with a mixtures of *Agrobacterium* cultures containing 35S-GFP plus the empty vector or 35S-GFP plus P19, served as negative or positive controls, respectively. Photographs were taken under UV light at 6 dpi. White arrows indicate the red ring, a hallmark of short-distance spread of the mobile RNA silencing signal at the edge of the infiltrated patches. (**B**) MMDaV V2 could interfere with systemic spread of RNA silencing signal in *N. benthamiana* 16c plants. The upper panels represent *N. benthamiana* 16c plants infiltrated with *Agrobacterium* cells carrying 35S-GFP plus empty vector (with systemic silencing), or 35S-GFP plus MMDaV V2 (with no systemic silencing), or 35S-GFP plus P19 (with no systemic silencing). Photographs were taken under UV light at 20 dpi. The number of *N. benthamiana* 16c plants systemically silenced at 20 dpi was indicated. *N. benthamiana* 16c plants that turned red in the major and minor veins of upper young leaves were considered to be systemically silenced.

In plants, after silencing is initiated in a single cell, the silencing signal can spread locally from cell to cell. To investigate whether the MMDaV V2 protein that was identified as a local silencing suppressor can inhibit the cell-to-cell spread of RNA silencing, we monitored GFP fluorescence at the edge of the infiltrated patches in *N. benthamiana* 16c plants. In this system, movement of the silencing signal from the agroinfiltrated area causes a strong reduction of GFP transgene expression in a zone of 10 to 15 adjacent cells, which can be visualized under UV light as a characteristic red ring around the infiltration area [36]. As expected, when the leaves of *N. benthamiana* 16c plants were co-infiltrated with 35S-GFP and the empty pCHF3 vector, a significant decrease of GFP expression and an obvious red ring was observed in the cells surrounding the agroinfiltrated patches at 6 dpi (Figure 4A). In contrast, no red ring was developed around the infiltrated zone expressing 35S-GFP and P19, as previously described (Figure 4A). Significantly, a visible red ring was observed around the infiltrated area expressing 35S-GFP and MMDaV V2 (Figure 4A). These results suggest that MMDaV V2 does not suppress the short range (cell-to-cell) spread of RNA silencing.

### 3.4. MMDaV V2 Inhibits Systemic Silencing of GFP

In plants, the silencing signal can also move systemically through the phloem. To find out the effect of MMDaV V2 on long-range movement of RNA silencing, GFP fluorescence was monitored in the newly emerging leaves of *N. benthamiana* 16c plants infiltrated with 35S-GFP and the empty pCHF3 vector, or 35S-GFP and V2, or 35S-GFP and P19. At 20 dpi, red fluorescence was observed in upper uninfiltrated leaves of 35S-GFP and empty vector-infiltrated plants (16 out of 24 plants showed red fluorescence), an indication of systemic silencing (Figure 4B). In contrast, upper leaves of *N. benthamiana* 16c plants infiltrated with 35S-GFP plus MMDaV V2 or P19, maintained GFP fluorescence. As shown in Figure 4B, the efficiency of MMDaV V2 to inhibit systemic silencing was 87.5% (21 of 24 plants showed inhibition of systemic silencing), indicating that MMDaV V2 was able to inhibit long-distance spread of RNA silencing.

### 3.5. The Basic Motif Is Required for MMDaV V2 Subnuclear Foci Localization and PTGS Suppression

**Figure 5:**
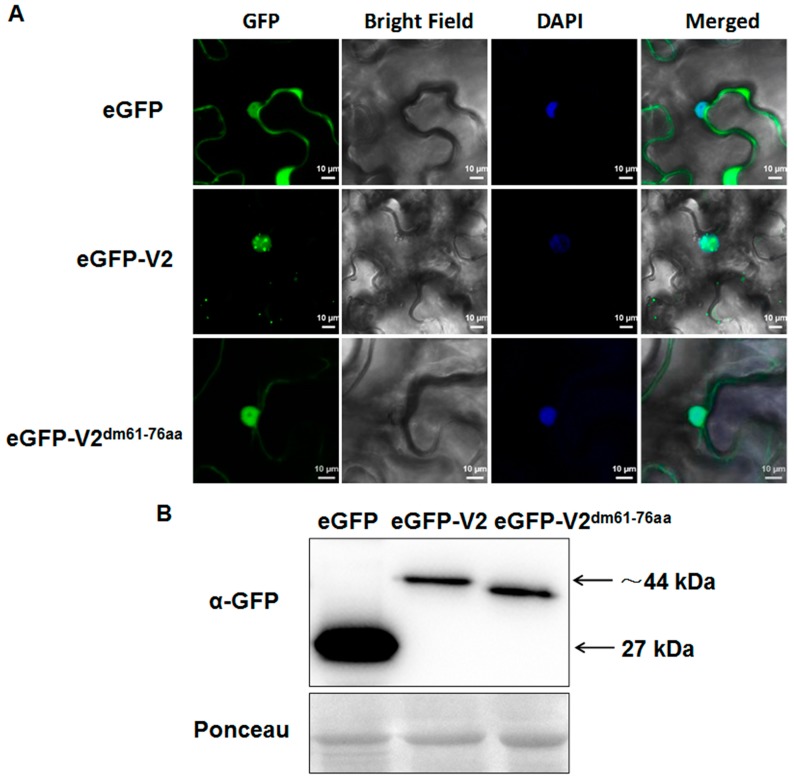
Deletion of the basic motif of MMDaV V2 fails to localize to subnuclear foci. (**A**) Subcellular localization of MMDaV V2 and V2 mutant variant in epidermal cells of *N. benthamiana*. *Agrobacterium* cells containing 35S-eGFP, 35S-eGFP-V2, 35S-eGFP-V2^dm61-76aa^ was infiltrated into leaves of *N. benthamiana*, respectively. DAPI staining was used to visualize the nuclei. White bars represent 10 µm. All images were visualized by confocal microscopy (Zeiss LSM880) at 36 to 48 h post-infiltration. Independent infiltration experiments were performed three times, and 5–6 cells were examined in each experiment. (**B**) Gel blot of total proteins from *N. benthamiana* leaves infiltrated with constructs represented in (**A**) using anti-GFP antibody. Ponceau staining serves as a loading control.

To explore which region(s) within MMDaV V2 is crucial for suppressing RNA silencing, we analyzed the MMDaV V2 sequence, and found that the RRLLRLIRRFSRVKDR motif between amino acid 61 and 76 defined a bipartite basic nuclear localization signal (NLS). Previous studies have indicated that the NLS of several VSRs is essential for the RNA silencing suppressor activity [31,37,38,39], prompting us to determine whether this motif is crucial for V2 to suppress PTGS. At a first step, we determined the subcellular localization of V2 via transient expression of eGFP-fused constructs into *N. benthamiana* leaves. An examination of leaves expressing eGFP, as a control, by laser scanning confocal microscopy at 36 h postinfiltration showed that both eGFP and eGFP-V2 were localized in the nucleus and in the cytoplasm. A close-up of the images showed that expression of GFP-V2 localized to the subnuclear foci as indicated by DAPI staining (Figure 5A). Interestingly, the GFP-V2 fusion protein formed one or more discrete fluorescent foci inside the nucleus (Figure 5A).

To investigate whether the predicted NLS plays a role in protein localization, the 61RRLLRLIRRFSRVKDR76 motif was deleted from V2 and a cassette to express the V2 mutant variant, 35S-GFP-V2^dm61-77aa^, was generated. In contrast to the wild-type V2, the fluorescence of 35S-GFP-V2^dm61-77aa^ was generally distributed throughout the nucleus and the cytoplasm. Despite the observation that deletion of the putative NLS from MMDaV V2 did not prevent localization of the protein in the host nucleus, 35S-GFP-V2^dm61-77aa^ appeared not to form discrete foci in the plant nuclear (Figure 5A). To ensure that the inability of the 35S-GFP-V2^dm61-77aa^ to form the subnuclear foci was not due to destabilization or impaired production of the mutant V2 protein, Western blot analysis was carried out to detect the eGFP fusion proteins using anti-GFP antibody. Detection of the expected protein sizes revealed that the deletion of the 61RRLLRLIRRFSRVKDR76 motif does not destabilize or impair the production of the V2 protein (Figure 5B). Together, these results suggest that the basic motif of MMDaV V2 contains signals conditioning formation of the subnuclear foci in plant cells.

**Figure 6:**
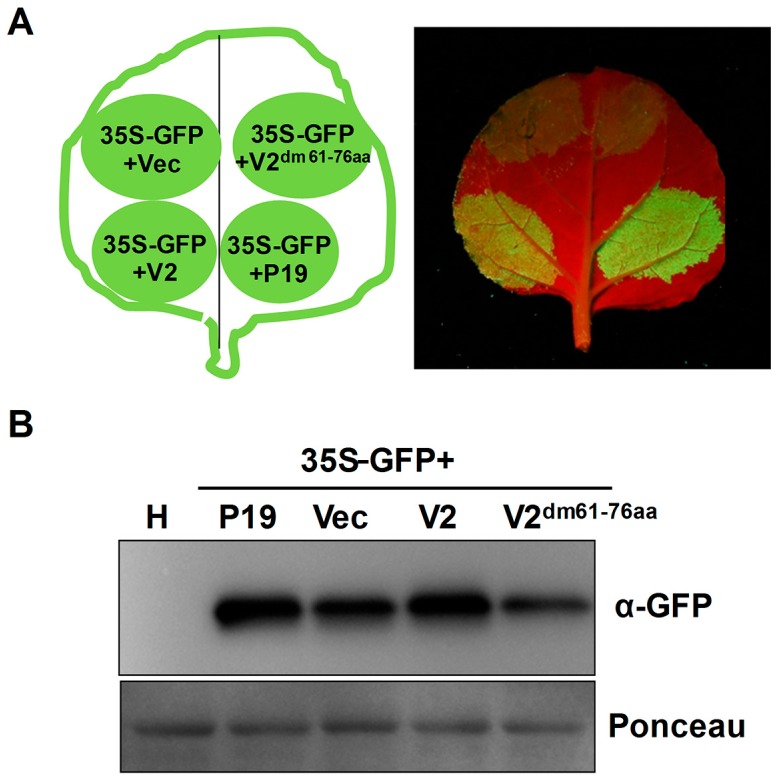
Deletion of the basic motif of MMDaV V2 influences its RNA silencing activity. (**A**) Suppression of RNA silencing in *N. benthamiana* plants. Leaf patches were infiltrated with a mixture of *Agrobacterium* cultures containing constructs represented in the left panel. Photographs were taken under UV light at 3 dpi. Three independent infiltration experiments were carried out, and four plants were used per experiment. (**B**) Western blot analysis of the GFP protein levels in infiltrated leaf patches using a monoclonal antibody against GFP. Total proteins extracted from the healthy *N. benthamiana* plant (H) were used to detect the specificity of the GFP antibody. Ponceau staining of the large subunit of Rubisco was used as loading controls.

To determine whether the basic motif of MMDaV V2 is required for its VSR activity, V2^dm61-77aa^ was cloned into pCHF3 to evaluate its ability to suppress RNA silencing. Leaves monitored under UV light showed that co-expression of 35S-GFP and empty vector or 35S-GFP and V2^dm61-77aa^ led to a loss of GFP fluorescence at 3 dpi (Figure 6A). This observation was further confirmed by examining the relative GFP protein level extracted from the corresponding leaf patches (Figure 6B), suggesting that the 61RRLLRLIRRFSRVKDR76 motif is required for MMDaV V2 to suppress RNA silencing. Taken together, these findings indicate that the 61RRLLRLIRRFSRVKDR76 motif was vital for V2 subnuclear foci localization and RNA silencing suppression.

## 4. Discussion

With limited coding capacity, plant viruses of the family *Geminiviridae* rely extensively on the host machinery for their infection cycles, such as replication and cell-to-cell and systemic movement. Geminiviruses therefore redirect and reprogram multiple plant processes to achieve a successful infection [27,40]. To combat a geminivirus infection, plants have employed sophisticated defense systems. The RNA silencing machinery, an antiviral defense conserved in plants and other eukaryotic organisms, is implicated as the major effective mean to overcome geminivirus infection [28]. As a counterdefense, geminiviruses evolve to encode silencing suppressors that target different steps of the RNA silencing pathway [27,28,41]. In this study, we screened for the potential virulence factors and RNA silencing suppressors encoded by MMDaV. We report that the V2, RepA, and Rep proteins of MMDaV affect the pathogenicity of PVX. We present evidence that MMDaV V2 is a strong suppressor of PTGS, and that it localizes to subnuclear foci and the cytoplasm. Finally, we show that the motif from amino acids 61 to 76 is crucial for MMDaV V2 to form subnuclear foci in plant cells and to suppress PTGS.

V2 from several Old World begomoviruses has been identified to have an RNA silencing suppressor activity [42,43,44,45,46,47,48]. The V2 protein of tomato yellow leaf curl virus (TYLCV) is proposed to suppress PTGS by direct interaction with suppressor of gene silencing 3 (SGS3) [47], or through competition with SGS3 for dsRNA substrates [49]. The V2 protein encoded by tomato yellow leaf curl China virus was shown to bind siRNAs [42]. In a recent study, V2 from a curtovirus is reported to act as a PTGS suppressor, possibly by hindrance of the RNA-dependent RNA polymerase 6 (RDR6) function [50]. Despite similarity in genome location, the length and amino acid sequence of MMDaV V2 are highly diverse from its begomovirus and curtovirus counterpart. In this study, it is clear from the co-infiltration leaf patch assays performed on *N. benthamiana* 16c or wild-type *N. benthamiana* plants that MMDaV V2 suppresses RNA silencing triggered by sense *GFP* RNA. In spite of the fact that MMDaV V2 could not prevent cell-to-cell spread of the silencing signal, MMDaV V2 could interfere with systemic spread of the silencing signal. As MMDaV V2 does not inhibit local silencing induced by dsRNA, it probably targets the upstream steps of dsRNA production. Sense GFP-triggered silencing requires the conversion to dsRNA by the action of RDR6, in concert with other co-factors, such as SGS3 [51,52,53]. It is not known whether MMDaV V2 suppresses PTGS by impairing the RDR6/SGS3 pathway. Failing in detecting an interaction between MMDaV V2 and SGS3 suggests that there may be mechanistic differences between MMDaV V2 and TYLCV V2 (Xiuling Yang, Dongxue Wang, Xueping Zhou. Unpublished. Institute of Plant Protection, Chinese Academy of Agricultural Sciences, Beijing, China. 2018). The mode of action and molecular targets of this divergent V2 protein remain to be elucidated.

It is interesting to note that, different from the V2 protein of Old World begomoviruses and beet curly top virus, the GFP-tagged MMDaV V2 protein localized both to subnuclear foci and the nucleus. In silico prediction of MMDaV V2 domains showed that it contains a bipartite NLS (RRLLRLIRRFSRVKDR) from amino acids 61 to 76. Despite the fact that deletion of the putative NLS from MMDaV V2 did not prevent translocation of the protein into the plant nucleus, mutagenesis experiment demonstrated that the predicted basic motif of MMDaV V2 is indispensable not only for subnuclear foci localization but also for silencing suppression. In previous studies, nuclear import was found mandatory for several VSRs, such as βC1 of tomato yellow leaf curl China betasatellite and tomato leaf curl China betasatellite, P37 of Pelargonium line pattern virus, and P6 of cauliflower mosaic virus (CaMV) and strawberry vein banding virus, to suppress RNA silencing [31,37,38,39,54]. Nuclear import of CaMV P6 is conducted via two importin-α-dependent NLSs, and it is required for CaMV infection and suppression of the nuclear RNA silencing factor dsRNA-binding protein 4 (DRB4) [54]. In the case of CMV 2b, the NLS sequence in the Fny2b is coincident with the domain required for sRNA binding [55]. Although earlier studies showed that nuclear enrichment of 2b was required for RNA silencing suppression, latter studies found that small RNA binding activity is required for VSR activity, while nuclear localization is dispensable [55]. The nuclear–cytoplasmic partitioning of the 2b protein allows CMV to regulate the balance between virus accumulation and suppression of RNA silencing [56]. At this moment, we do not know how the subnuclear foci localization of MMDaV V2 influences its VSR activity. Nonetheless, our results demonstrate that silencing suppression is one of several possible nuclear function of MMDaV V2.

VSRs generally have parallel functions. Besides working as silencing suppressors, they may also fulfill other non-silencing suppression tasks during infection. Therefore, VSRs can often differentially affect pathogenicity in heterologous systems. In agreement with previous studies [42,44], expression of MMDaV V2 by PVX induced leaf curling and a HR-like cell death symptoms that were distinct from the symptoms induced by PVX infection, suggesting that the MMDaV V2 protein might play some role in pathogenicity or virulence. Although the heterologous expression of MMDaV V2 led to an enhancement of symptoms leading to plant death at 11 dpi, decreased PVX accumulation was observed in non-inoculated *N. benthamiana* leaves when compared to those inoculated with PVX. It is generally thought that due to the suppression of antiviral defenses by VSRs, chimerical PVXs carrying heterologous VSRs accumulate at higher levels when compared with the wild-type PVX. However, previous studies indicated that the combination of PVX with some VSRs like P19 and HC-Pro that are capable of inducing a systemic necrosis response in *N. benthamiana* does not relate to an increase in the PVX genomic RNA levels, but enhances and/or stabilizes PVX subgenomic RNAs [57]. In the case of the heterologous expression of tomato chlorosis virus p22 suppressor, no obvious differences in PVX accumulation were observed in the viral accumulation of PVX-p22 and PVX [58]. Since the enhanced pathogenicity associated with recombinant PVX-V2 does not seem to link to a more efficient viral accumulation, other mechanisms must be involved. It is also remarkable that the areas infiltrated with 35S-GFP plus RepA or 35S-GFP plus Rep became necrotic at three to four dpi in the co-infiltration leaf patch assays, suggesting that RepA and Rep may be associated with other plant defenses.

The disease denoted mulberry mosaic dwarf (MMD) has seriously affected *Morus alba* in China for a long time, and it has a close association with MMDaV. Our findings present the first identification of a VSR from a woody plant-infecting geminivirus, providing a starting point for understanding the mechanisms that are involved in MMDaV-induced MMD.

## Figures and Tables

**Table 1:** Synthetic oligonucleotide primers used in this study.

Primers	Sequence (5′–3′)
**Primers used for the construction of recombinant PVX vector or pCHF3-based binary vectors**	
V1/*Sma*I *Cla*I-F	CCCGGGATCGATatggtgattaccaggagctc
V1/*Sal*I-R	GTCGACttattctgcgtcataaaaataaac
V2/*BamH*I *Cla*I-F	GGATCCATCGATatgtctttgtggagtaccaaattag
V2/*Sal*I-R	GTCGACttaattccaaatgtgccacg
V3/*BamH*I *Cla*I-F	GGATCCATCGATatgagctataaatacccccctgc
V3/*Sal*I-R	GTCGACctacggcactgagtaaggtg
V4/*Kpn*I *Cla*I-F	GGTACCATCGATatgttttcaaggagaaaaaaag
V4/*Sal*I-R	GTCGACctagtttattacatgtctgctag
V5/*Kpn*I *Cla*I-F	GGTACCATCGATatgccggaagctctcgacgattg
V5/*Sal*I-R	GTCGACctaatctcctctgcgtttctttaag
C1C2/*BamH*I *Cla*I-F	GGATCCATCGATatggcttcaagttctaacttcag
RepA/*Sal*I-R	GTCGACctaaagatctggcccattgc
Rep/*Sal*I-R	GTCGACttaatagaatttatcactagcagac
**Primers used to generate V2 mutant**	
V2^dm61-76aa^/F	gctgcagtaaatggtgattaccagg
V2^dm61-76aa^/R	gcactgagtaaggtggaccaagtgg

## Supplementary Materials

The following are available online at http://www.mdpi.com/1999-4915/10/9/472/s1. Figure S1. The effect of MMDaV ORFs on the prevention of GFP silencing. *Nicotiana benthamiana* 16c plants were infiltrated with a mixture of *Agrobacterium* cultures containing 35S-GFP and pCHF3 vectors expressing individual ORFs of MMDaV, respectively. The constructs used for infiltration are indicated. Photographs were taken under UV light with a yellow filter-mounted Canon camera or under natural light at 8 dpi. Note that co-infiltration of 35S-GFP with RepA or Rep caused necrotic lesions in infiltrated areas at 8 dpi. Figure S2. MMDaV V2 does not suppress dsRNA-induced RNA silencing. *N. benthamiana* 16c plants were infiltrated with a mixture of *Agrobacterium* cultures containing 35S-GFP, 35S-dsGFP and MMDaV V2. Co-infiltration of *N. benthamiana* 16c plants with 35S-GFP, 35S-dsGFP and the empty pCHF3 vector or P19 served as negative or positive controls, respectively. Photographs were taken under UV light at 3 dpi.
