# Supplementary material for: Identification of the Potential Virulence Factors and RNA Silencing Suppressors of Mulberry Mosaic Dwarf-Associated Geminivirus
Source: Viruses. 2018 Sep 3;10(9):472. doi: 10.3390/v10090472 (PMC6163789; doi:10.3390/v10090472)
Supplement: Supplementary file 1 [file viruses-10-00472-s001.pdf]

Supplementary File

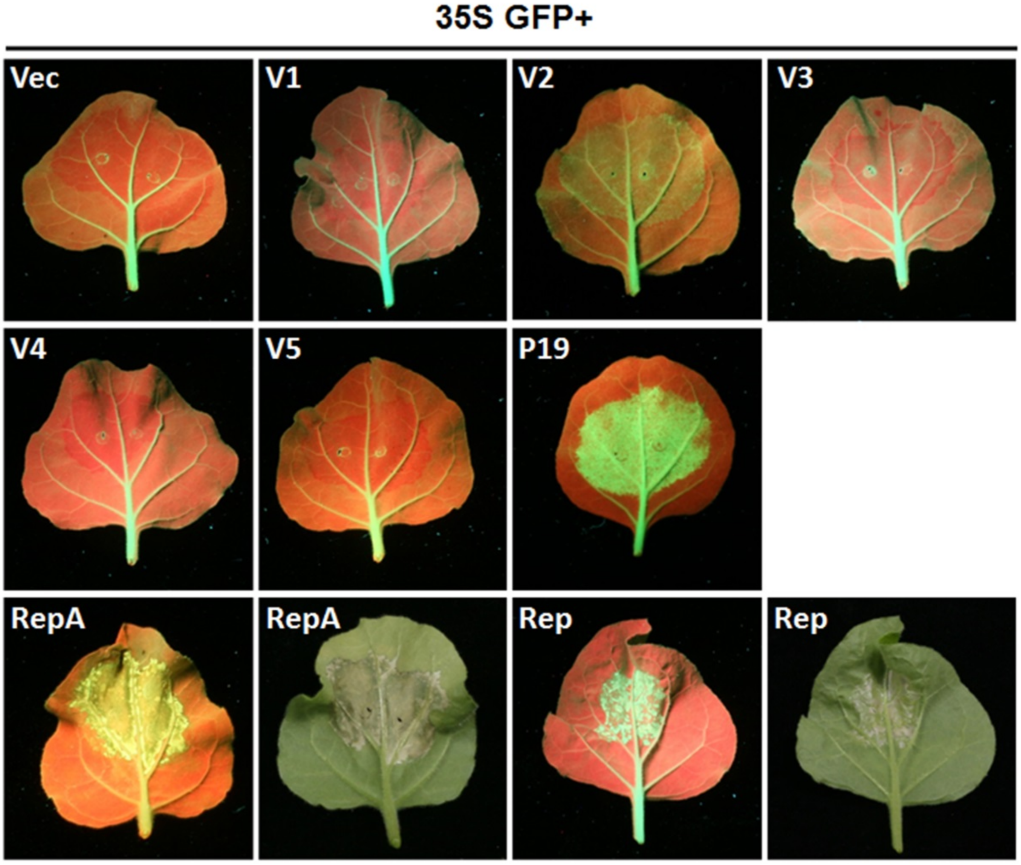

Figure S1. The effect of MMDaV ORFs on the prevention of GFP silencing. *Nicotiana benthamiana* 16c plants were infiltrated with a mixture of *Agrobacterium* cultures containing 35S-GFP and pCHF3 vectors expressing individual ORFs of MMDaV, respectively. The constructs used for infiltration are indicated. Photographs were taken under UV light with a yellow filter-mounted Canon camera or under natural light at 8 dpi. Note that co-infiltration of 35S-GFP with RepA or Rep caused necrotic lesions in infiltrated areas at 8 dpi.

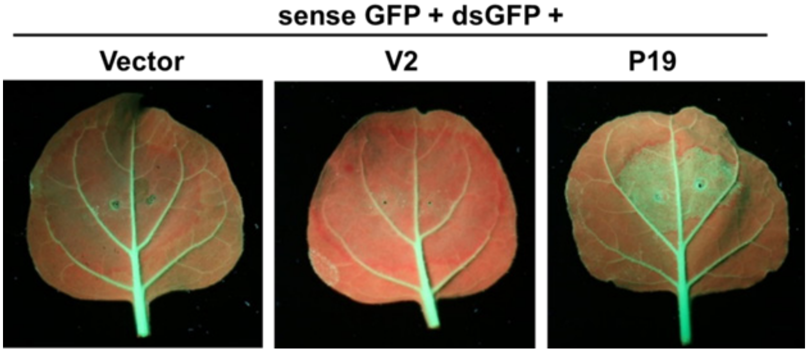

Figure S2. MMDaV V2 does not suppress dsRNA-induced RNA silencing. *N. benthamiana* 16c plants were infiltrated with a mixture of *Agrobacterium* cultures containing 35S-GFP, 35S-dsGFP and MMDaV V2. Co-infiltration of *N. benthamiana* 16c plants with 35S-GFP, 35S-dsGFP and the empty pCHF3 vector or P19 served as negative or positive controls, respectively. Photographs were taken under UV light at 3 dpi.
